# Unnatural Causes: Is Inequality Making Us Sick?

**Published:** 2007-09-15

**Authors:** Larry Adelman

**Affiliations:** California Newsreel

**Figure:**
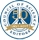
This article is part of PCD’s participation in the Council of Science Editors’ Global Theme Issue on Poverty and Human Development. Other PCD articles in this series are: Health, Wealth, and Well-Being

*Unnatural Causes*, a documentary series scheduled for release this fall, will present for the first time on American television an investigation of the sources of our huge and alarming socioeconomic and racial disparities in health. The seven-part, 4-hour documentary series will be available on DVD from California Newsreel (www.newsreel.org) and will be broadcast in the United States by Public Broadcasting Service (PBS) during the first half of 2008.

The video clip linked on this page offers a "sneak peek" at the series' opening episode, a look at what filmmakers call a "rough cut" of the work in progress. *Unnatural Causes* sifts through the evidence suggesting that there is more to our health than bad habits, problems with the health care system, or unlucky genes, and that the social environments in which we are born, live, and work profoundly affect our well-being and longevity.

Conceived as part of a larger public engagement campaign in association with leading public health, policy, and community-based organizations, *Unnatural Causes* aims to stimulate a national dialogue about what we as a society should and can do to address our health inequities. Although hundreds of studies investigate the pathways through which racial and socioeconomic status affect health, until now virtually no popular media — print, television, or Web — have translated this research into forms that can build public understanding of how social policies are de facto health measures. As a result, the common-sense wisdom remains that minorities and the poor get sick because they have unlucky genes or that they are just too lazy and undisciplined to eat right, exercise, and abstain from drugs and alcohol. Similarly, it is still widely believed that it is top executives who are dropping dead from heart and artery disease, when in truth it is their subordinates.

*Unnatural Causes* sets out to change that. It not only sounds the alarm about the extent of our health inequities but also explores how socioeconomic status and racism may be involved. Why do members of some populations get sicker more often? Through what channels might inequality — the cumulative disparities in housing, wealth, jobs, and education — combined with a lack of power and control over one's life translate into bad health? What is it about our poor neighborhoods, especially poor neighborhoods of color, that is so deadly? How are the lifestyle choices we make (e.g., diet, exercise) constrained by the choices we have?

*Unnatural Causes* does not dismiss the role individuals can play. On the contrary, healthy behaviors are critical. But they are only one part of the picture. As Harvard sociologist David Williams points out in the series, "Housing policy is health policy; educational policy is health policy; neighborhood improvement policies are health policies. Everything we can do to improve the quality of life of individuals in our society has an impact on their health and is a health policy." These are the stories *Unnatural Causes* tells.

**Figure:**
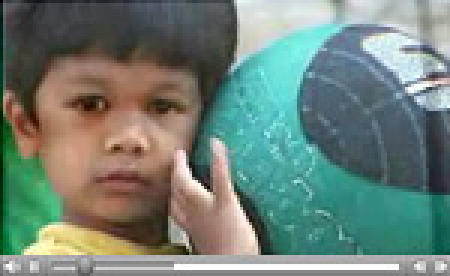

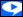
Watch a clip from *Unnatural Causes* (RM 1.9mb) You will need RealOne Player to view these videos. Learn more about RealPlayer. Learn more about RealPlayer.

**Table:** 

NARRATOR:
Living in America should be a ticket to good health. We have the highest gross national product in the world.
GORDON STAUFFER:
It’s good to have some of my cars in one place. . . .
SIR MICHAEL MARMOT:
If you look at the U.S. as a whole, the U.S. is the second richest country in the world after Luxembourg.
NARRATOR:
We have the most sophisticated medical technology. The most Nobel laureates in medicine. We spend 1.7 trillion dollars per year on medical care. That’s nearly half of all the health dollars spent in the world.
*Slowly scroll down first five countries on UNDP list of life expectancy and infant mortality statistics.*
NARRATOR:
Shouldn’t we rank at the top of international health indicators?
*Scroll down the next five.*
NARRATOR:
Or at least in the top five? The top ten?
*Pick up pace through the next 20 countries until we come to the United States.*
NARRATOR:
We barely make it among the top 30. We’re 30th in life expectancy. Costa Ricans live longer.
DAVID WILLIAMS:
Especially of the similar economically developed countries, we are at the bottom of the list.
NARRATOR:
A higher percentage of our babies die in their first year of life than in Cuba, Slovakia, or Estonia.
NARRATOR:
And this is not because of our so-called “melting pot.”
DR. RICHARD DAVID:
White Americans, if they were a separate country, would still rank 23rd in the world.
NARRATOR:
How can this be?
SIR MICHAEL MARMOT:
There are huge inequalities in the society. All this wealth is maldistributed.
NARRATOR: :
Inequality in America is higher today than at any time since before the Great Depression. And it’s greater than in any other industrialized nation.
SIR MICHAEL MARMOT:
And I think that’s in part why the U.S. as a whole has relatively poor health amongst the rich countries. And we think that that is not inevitable.
*Fade to black.*
PERSON:
text
NARRATOR:
This is a story about health in one American city. It’s not about health insurance or better drugs. But it is about how social conditions and policies can dictate who will be sicker, who will live longer. To see how that happens, you only have to stop and look, wherever you are. Even here.
WOMAN #1:
Welcome to Louisville.
WOMAN #2:
Welcome to Louisville.
MAN #1:
Welcome to Louisville.
MAN #2:
Louisville.
MAN #3:
Louisville.
MAN #4:
Louisville.
MAN #5:
Welcome to Louisville
NARRATOR:
Sixteenth largest city in the United States. Population 750,000.
DR. ADEWALE TROUTMAN:
Diabetes, cardiovascular disease, low birth weight, infant mortality.
DR. ADEWALE TROUTMAN:
Certainly, the health is bad. So there are major health problems in Louisville.
NARRATOR:
Overall, we are healthier than we were 50 years ago. But those gains are not shared equally. Dr. Adewale Troutman should know. As the director of public health, he can tell you that in some Louisville neighborhoods people die on average 13 years sooner than others. And he wants to know why.

The opening hour-long episode gives an overview of the problem. Taking University of British Columbia economist Robert G. Evans' metaphor of our medical system as our repair shop, where we go when our bodies break down, the episode asks what is causing so much wear and tear on our bodies, and why is that wear and tear so differentially distributed by race and class? How do social policy and the way we organize work and society affect health? Long-term solutions, the show suggests, lie not in more pills but in more equality.

The opening episode is supported by six additional 25-minute episodes set in different racial and ethnic communities. Each episode deepens understanding of the root causes of disease, demonstrates pathways through which social conditions may affect physiology, and brings viewers face-to-face with innovative initiatives for health equity.

One of the distinguishing features of *Unnatural Causes* is that the series was developed as part of a wider-impact campaign that calls for new prescriptions to tackle health inequities. As the result of work conducted by an outreach coordinating committee, organizations around the country have learned about the series and are putting plans in place to use it to inject social and economic policy — involving jobs, housing, labor policy, race, community development, education, and tax policy — into discussions of health and to evaluate social and economic policies by their impacts on health. Members of these groups are organizing briefings for public officials, teach-ins, training sessions, and community forums to educate constituents and build multi-sectoral alliances to improve community health by addressing "upstream" causes of poor health. One example of activities will involve a minimum of 100 public health departments throughout the country conducting community dialogues built around the series that bring together stakeholders from various nonhealth sectors. These public health departments are being supported by the National Association of County and City Health Officials (NACCHO). 

A toolkit on the series' companion Web site will support these impact campaign activities with planning and discussion guides, "myth-busting" video clips, community stress tests, and action suggestions. To learn more about the series and the community engagement campaign, visit www.unnaturalcauses.org.

